# The National and Global Impact of Systemic and Structural Violence on the Effective Prevention, Treatment, and Management of COVID-19 in African or Black Communities: Protocol for a Scoping Review

**DOI:** 10.2196/40381

**Published:** 2022-10-17

**Authors:** Roberta Timothy, Robert Ainsley Chin-see, Julia Martyniuk, Pascal Djiadeu

**Affiliations:** 1 Dalla Lana School of Public Health University of Toronto Toronto, ON Canada; 2 Gerstein Science Information Centre University of Toronto Toronto, ON Canada; 3 Health Research Methods Evidence and Impact McMaster University Hamilton, ON Canada

**Keywords:** African, Black people, COVID-19, systemic and structural barriers, health disparities, minority health, racism, racial health inequity, structural violence, anti-Black racism, decolonizing, resistance, social justice

## Abstract

**Background:**

As COVID-19 ravages the globe and cases increase rapidly, countries are presented with challenging policy choices to contain and mitigate its spread. In Canada and globally, the COVID-19 pandemic has added a new stratum to the debate concerning the root causes of global and racial health inequities and disparities. Individuals who exist as targets of systemic inequities are not only more susceptible to contracting COVID-19, but also more likely to bear the greatest social, economic, and physical burdens. Therefore, data collection that focuses on the impact of COVID-19 on the lives and health of African/Black communities worldwide is needed to develop intersectional, culturally relative, antiracist/antioppression, and empowerment-centered interventions and social policies for supporting affected communities.

**Objective:**

The primary objective of this review is to investigate the impact and management of COVID-19 among African/Black individuals and communities, and understand how anti-Black racism and intersectional violence impact the health of African/Black communities during the pandemic. Moreover, the study aims to explore research pertaining to the impact of COVID-19 on Black communities in the global context. We seek to determine how Black communities are impacted with regard to structural violence, systematic racism, and health outcomes, and the ways in which attempts have been made to mitigate or manage the consequences of the pandemic and other injurious agents.

**Methods:**

A systematic search of quantitative and qualitative studies published on COVID-19 will be conducted in MEDLINE (Ovid), Embase (Ovid), Cumulative Index to Nursing and Allied Health Literature (EBSCO), Cochrane Library, PsychInfo (Ovid), CAB Abstracts (Ovid), Scopus (Elsevier), Web of Science (Clarivate), and Global Index Medicus. To be included in the review, studies should present data on COVID-19 in relation to African/Black individuals, populations, and communities in the global sphere. Studies must discuss racism, oppression, antioppression, or systemic and structural violence and be published in English, French, Spanish, or Portuguese. According to the Preferred Reporting Items for Systematic Reviews and Meta-Analyses extension for Scoping Reviews guidelines, the findings will be synthesized quantitatively and qualitatively through thematic analysis. The risk of bias will not be assessed.

**Results:**

Title, abstract, and full-text screening concluded in June 2022. Data collection is in progress and is expected to be completed by December 2022. Data analysis and drafting of the manuscript will be done thereafter. Findings from the scoping review are expected to be provided for peer review in 2023.

**Conclusions:**

This review will collect important data and evidence related to COVID-19 in African/Black communities. The findings could help identify existing gaps in COVID-19 management in African/Black communities and inform future research paradigms. Furthermore, the findings could be applied to decision-making for health policy and promotion, and could potentially influence services provided by health care facilities and community organizations around the globe.

**International Registered Report Identifier (IRRID):**

DERR1-10.2196/40381

## Introduction

As SARS-CoV-2 ravaged the globe and the number of cases increased rapidly, countries were presented with challenging policy choices to contain the spread of COVID-19. Although this was a global effort, each country had a different response to the pandemic. For example, some countries, such as China and South Africa, elected to immediately close the national economy and apply strict and punishable rules regarding traditional public health measures (eg, social distancing, masking, isolation, and quarantine) [1]. In contrast, other countries, including the United States, opted for more loose public health recommendations. Although research has found that public health interventions and nonpharmaceutical control measures are effective in mitigating transmission of COVID-19, the differential timing of lockdown measures, including closing nonessential industries and limiting in-person capacity, may have significant social and economic implications [2,3]. One of the potential concerns is how the COVID-19 pandemic has differentially affected populations, as it appears that the burden of these interventions was not equal. The COVID-19 pandemic has created a wave of panic across the world. According to John Hopkins University, COVID-19 has taken the life of 4,310,354 people across the globe, with 26,633 deaths in Canada alone as of August 10, 2021 [4]. The COVID-19 pandemic has added a new stratum to the debate concerning the root causes of racial health disparities. The effects of COVID-19 have been shown to be linked to structural violence and racism [5]. The British and American governments have acknowledged that large proportions of their COVID-19 cases and deaths are among individuals of African descent. Due to the discrimination and oppression experienced by racialized groups, such as Black communities, it was reported that in England, Black people were more than 4 times likely to die from COVID-19 than their White counterparts [5,6]. Similarly, an analysis by the Washington Post reported that in the United States, counties where Black residents were in majority had 3 times the rate of COVID-19 infection and almost 6 times the rate of deaths compared with counties where White residents were in majority [7].

Multiple researchers have misattributed the morbidity and mortality disparities observed in England and America to the high prevalence of chronic diseases in Black communities [8,9]. Multinational data have reported poor outcomes for individuals aged over 65 years or with underlying health conditions, including diabetes, heart disease, asthma, and compromised immune systems [10].

The association between COVID-19 and pre-existing illness is especially troubling for Black individuals who are genetically misconceived as more likely to develop chronic comorbidities because they are Black [11].

Many social determinants of health, including anti-Black racism, intersectional violence (including sexism, heterosexism, classism, ageism, refugee status, etc), poverty, physical environment (eg, smoke exposure and homelessness), and race and ethnicity, can have a considerable effect on COVID-19 outcomes. Homeless families are at higher risk of viral transmission because of crowded living spaces and scarce access to COVID-19 screening and testing facilities [12].

Individuals who exist as targets of systemic inequities are not only more susceptible to contracting COVID-19, but also more likely to bear the greatest extent of the subsequent economic pandemic [13].

There is a need for data collection that specifically focuses on the impact of COVID-19 on the lives and health of African/Black communities across the globe in order to develop intersectional, culturally relative, antiracist/antioppression, and empowerment-centered interventions and social policies that support heterogeneous African/Black communities during and after the COVID-19 pandemic.

The collection of race-based data on COVID-19 is important to understand the impact of COVID-19 on the lives of African/Black people and its historical and current day context [14].

In most African countries, the response to the COVID-19 pandemic has been challenging due to continued colonial impacts, which lead to distrust in the government, and social, cultural, and religious resistance [15]. The COVID-19 global pandemic has exposed the world inequities and the racially based colonial demarcations with the North/South as the main geographical and sociological anchors [16]. In Brazil, the failure of the neoliberal government to protect the Black and Indigenous populations mostly exposed to COVID-19, has created the emergence of a new form of solidarity and mutual aid in “favelas” and Indigenous communities [17].

Standard systematic reviews and scoping reviews are different; scoping reviews investigate broad topics as opposed to a specific well-defined question. In the context of this paper, this review will assess the impact of COVID-19 on African/Black communities across the globe. The purpose of this scoping review is to employ a decolonizing, African feminist, Black resistance lens to examine the impact of COVID-19 on heterogeneous and intersectional Black communities throughout the world, while also exploring the various forms of resistance that Black communities have established and employed during the global pandemic. Therefore, this review will attempt to answer the question of how COVID-19 is impacting African/Black communities, and what interventions are effective to prevent, treat, and reduce the impact of COVID-19 on these communities?

The primary objectives are to (1) investigate the impact of COVID-19 on African/Black individuals and communities; (2) explore how systemic and structural violence are barriers to the effective prevention, treatment, and management of COVID-19 in the African/Black population; and (3) understand how anti-Black racism and intersectional violence (violence related to race, gender, sexual orientation, gender identity, age, disabilities, language, educational attainment, immigration status, and social determinants of health) impact the health of African/Black communities during the COVID-19 pandemic. The secondary objective of the review is to identify intervention strategies to respond effectively to the impact of COVID-19 on African/Black individuals, communities, front-line health service workers, and essential service workers in Canada and transnationally.

## Methods

### Approach

A scoping review methodology has been selected as it can help to (1) identify review parameters, (2) identify a process of mapping the existing literature, and (3) explore a research gap [18].

The methodological framework as described by Arksey and O’Malley, and later advanced by Levac et al will be applied and followed in developing and disseminating this scoping review [19,20]. The scoping review approach will also endeavor to include recommendations by Peterson that are appropriate for policy change, education, and research purposes [21]. The protocol has been drafted with the intention to align with the reporting guidance provided in the Preferred Reporting Items for Systematic Reviews and Meta-Analyses extension for Scoping Reviews (PRISMA-ScR) checklist (Multimedia Appendix 1) [22-24]. This framework recommends organizing the scoping review in a 6-stage process that includes but is not limited to (1) identifying the research question; (2) identifying relevant studies; (3) selecting studies; (4) charting the data; (5) collating, summarizing, and reporting the results; and (6) consulting with relevant stakeholders and key informants.

### Identifying the Research Question

The research question was developed in response to a knowledge gap in the literature surrounding the health of Black people in relation to the COVID-19 pandemic, and revised periodically with significant input from the principal investigator. The following research question was identified and guided the scoping review and effective search strategy: How is COVID-19 impacting African/Black communities nationally and globally, and what interventions are effective to prevent, treat, and reduce the impact of COVID-19 on these communities?

### Identifying Relevant Studies

An expert liaison and education librarian familiar with systematic and scoping review processes was enlisted to assist with devising the search strategy and other essential resources. The research team conducted a preliminary assessment of a variety of electronic databases independently, such as PubMed and Google Scholar, using search terms associated with the domains of interest. Reference lists of articles deemed relevant were scanned much like their titles and abstracts, which culminated in many articles. On that basis, keywords were collected and used in the search strategy, and we found a need to expand the geographic inclusion beyond Canada.

To that extent, a comprehensive systematic search was developed and employed in multiple electronic databases: MEDLINE (Ovid), Embase (Ovid), Cumulative Index to Nursing and Allied Health Literature (EBSCO), Cochrane Library, APA PsychInfo (Ovid), CAB Abstracts (Ovid), Scopus (Elsevier), Web of Science (Clarivate), and Global Index Medicus. This electronic search strategy was developed by a health science librarian (JM) and was peer reviewed according to the peer review of electronic search strategies (PRESS) guidelines [25]. The literature search systematically searched the published literature of quantitative and qualitative studies published since the beginning of COVID-19 in December 2019, and will be documented in accordance with the PRISMA-ScR checklist [23].

### Types of Studies

Experimental (randomized or nonrandomized), observational (longitudinal or cross-sectional), qualitative, and mixed methods studies will be considered for this review.

### Eligibility Criteria

To be included in the review, studies should include data on COVID-19 in relation to African/Black individuals, populations, and communities, no matter the geographical status. The studies must discuss racism, oppression, antioppression, or systemic and structural violence, and be published in English, French, Spanish, or Portuguese. The research team includes members who are fluent in the aforementioned languages and will independently assess study articles to ensure relevant data can be extracted. Additionally, if non-English articles provide an English translation, this will be accepted so long as other criteria are met. Screening will be conducted to filter out studies based on their publication date and language. In particular, studies published between December 2019 and August 2021 will be included for review. Review papers (eg, scoping reviews, systematic reviews, and rapid reviews), reports, book chapters, and conference abstracts will be excluded from the review.

### Outcomes

With regard to the primary outcomes of this scoping review, we will (1) evaluate COVID-19 prevention, infection, testing, comorbidity and mortality, and interventions in African/Black individuals and communities, as well as the global impact on these communities; (2) investigate the effect of systemic and structural barriers on the prevention, treatment, and management of COVID-19 in a population that has been reported to experience significantly higher COVID-19 complications and negative outcomes; and (3) evaluate the impacts of social determinants of health and the intersections of dimensions, such as gender, sexual orientation, gender identity, age, disabilities, language, educational attainment, and immigration status, on COVID-19 in African/Black communities.

With regard to the secondary outcomes, we will assess (1) the resistance of African/Black communities in relation to the structural barriers that they face in the context of COVID-19 and (2) the population-based intervention strategies and tools to better prevent, treat, and manage COVID-19 in African/Black individuals and communities globally.

The search strategy can be found in Table 1, and it includes the concepts COVID-19, Black people, and racism. The search strategies for all the databases are detailed in Multimedia Appendix 2 and can also be found online [26].

**Table 1:** Proposed search strategy for MEDLINE (search on August 4, 2021).

#	Search	Results, n
1	exp Coronavirus/	87,181
2	exp Coronavirus Infections/	106,533
3	(coronavirus* or corona virus* or OC43 or NL63 or 229E or HKU1 or HCoV* or ncov* or covid* or sars-cov* or sarscov* or Sars$coronavirus* or Severe Acute Respiratory Syndrome Coronavirus* or 2019$nCov or Severe Acute Respiratory Syndrome Corona Virus).tw,kf,ot.	173,610
4	((novel or new or nouveau) adj2 (CoV or nCoV or covid* or coronavirus* or corona virus or Pandemi*)).tw,kf,ot.	14,611
5	((Wuhan or Hubei) adj5 pneumonia).tw,kf,ot.	355
6	((new or novel or “19” or “2019” or Wuhan or Hubei or China or Chinese) adj3 (coronavirus* or corona virus* or betacoronavirus* or CoV or HCoV)).tw,kf,ot.	48,596
7	((coronavirus* or corona virus* or betacoronavirus*) adj3 (pandemic* or epidemic* or outbreak* or crisis)).tw,kf,ot.	8997
8	1 or 2 or 3 or 4 or 5 or 6 or 7	185,346
9	limit 8 to yr=“2019 -Current”	166,753
10	exp african continental ancestry group/ or ethnic groups/	150,584
11	Minority Groups/	15,130
12	Minority Health/	839
13	(people of colo$r or person* of colo$r or POC or BAME or BIPOC or ((african* or afro*) adj5 (americ* or canad* or asia* or caribbean* or australi* or european* or brazil* or minorit* or refugee or migrant* or immigrant* or ancest* or native* or hispanic* or latin* or indigenous* or diaspora* or communit* or descen* or provider* or nurse* or doctor* or worker* or service user* or patient* or front line* or frontline* or people* or man or men or wom$n or race or population* or person* or individual* or group* or female* or male*))).tw,kf.	97,176
14	((black or blacks) adj5 (americ* or canad* or asia* or caribbean* or australi* or european* or brazil* or minorit* or refugee or migrant* or immigrant* or ancest* or native* or hispanic* or latin* or indigenous* or diaspora* or communit* or descen* or provider* or nurse* or doctor* or worker* or service user* or patient* or front line* or frontline* or people* or man or men or wom$n or race or population* or person* or individual* or group* or female* or male*)).tw,kf.	57,326
15	(((ethnic* or racial* or race) adj5 (group* or minorit* or disparit* or divers* or equal* or inequal* or discriminat*)) or mixed race or mixed racial* or multi racial* or mutli race or multiracial* or multirace).tw,kf.	87,613
16	10 or 11 or 12 or 13 or 14 or 15	286,281
17	prejudice/ or racism/	28,679
18	(racism or racist* or racial* or anti-black* or antiblack* or structural violence* or systemic violence*).tw,kf.	54,130
19	(white supremac* or white hegemon*).tw,kf.	95
20	(prejudice* or discriminat* or intolerance* or oppress* or bias* or hostil*).tw,kf.	550,272
21	((structur* or institution* or systemic or systematic* or generational* or intersect* or health*) adj5 (violence* or polic* or barrier* or disparit* or inequalit* or trauma)).tw,kf.	145,097
22	(decoloni* or de coloni* or anti oppress* or antioppress*).tw,kf.	1798
23	17 or 18 or 19 or 20 or 21 or 22	744,913
24	9 and 16 and 23	1349

### Study Selection

Search results will be deduplicated in EndNote before being uploaded to Covidence (JM), an online program that facilitates screening and data extraction [27].

Two reviewers will pilot test a screening form customized to reflect the aforementioned inclusion criteria. This screening form will be generated and used by 2 independent reviewers. A subset of records will be used as a sample to establish consistency of use and clarity of the instrument before its implementation. The Cohen kappa statistic will be estimated to measure interrater reliability, and screening will begin when 90% agreement is achieved [28].

With regard to study selection, initially, we will conduct title and abstract screening. Once an article is seen as potentially relevant, we will retrieve and screen the full text in detail. This occurs prior to data extraction. In duplicate, the authors RCS and PD will conduct all screening, data extraction, and quality assessment procedures. Disagreements will be resolved by consensus. If consensus cannot be reached, a third author (RT) will arbitrate.

The reference lists of all relevant citations will be searched for available related articles. We will search for available theses and conference posters. Furthermore, experts, authors, and relevant organizations will be contacted.

### Charting the Data

For the purpose of this scoping review, we will extract bibliometric information, such as author names, journal, and year of publication, in addition to the location of the study, study design, number of participants, outcomes reported, and outcome measures. We will report where possible measures of the effect of the outcome on African/Black people with respect to COVID-19. We will not report measures of magnitude, such as mean (SD) and percentage (95% CI), or extract data, such as odds or risk ratios and mean differences.

### Collating, Summarizing, and Reporting the Results

Our findings will be reported according to the PRISMA-ScR guidelines [23,29,30]. Our findings will be summarized narratively and using tables. Data will be grouped by outcomes, with the number of studies, their design, and their methodological quality. The key findings of each study will also be summarized using tables. We will conduct a narrative synthesis of the data to identify common themes and knowledge gaps. Since the review explores a variety of geographical regions across the globe, the results will be categorically interpreted based on common geographical demarcations (ie, North America, Latin America, Europe, Africa, Middle East, and Caribbean), grouped thematically, and compared. Similarly, data presentation will also include visualization on a geographical map to report the number of publications across the globe.

Throughout the reporting phase, we will follow the recommendations of the PRISMA-ScR guidelines when writing up the final review. Abstracted information from all the included articles will be synthesized, and the results will be presented to capture the extent of the literature. First, tables will provide basic information on the included articles, such as study characteristics, methodological quality, major findings, and the pedagogical and theoretical or conceptual approach used in the design. This overview will be followed by a narrative presentation of the synthesized mapping of the included literature. We will then report the output with an emphasis on describing how the findings relate to the research questions guiding the scoping review before discussing the implications for policy and practice in the realm of the health and well-being of Black people.

Consistent with the guidance on conducting scoping reviews, we will not assess the methodological quality or risk of bias of the included articles. Our goal is to provide an overview of the existing evidence and gap regardless of study quality.

### Consultation With Stakeholders

Patients are not involved in the design of the scoping review. Experts in the health and well-being of Black people may be consulted for informing the scoping review and disseminating research findings during presentation, but this is not expected.

### Ethical Considerations

Ethics approval is not required as secondary published data will be used.

## Results

This project started in August 2021 with the development of the search strategy and literature search. As of June 2022, the study team has completed title, abstract, and full-text screening of imported citations. Data collection is in progress and is expected to be completed by December 2022. Data analysis and drafting of the manuscript will be done thereafter, with expected publication in 2023. All data generated or analyzed during this study will be included in the published article and its supplementary information files.

## Discussion

### Overview

The primary goal of this review is to investigate the global impact of COVID-19 on African/Black individuals and communities, and explore how systemic and structural violence are barriers to the effective prevention, treatment, and management of COVID-19 in these communities.

The global COVID-19 pandemic is still occurring more than a year after its initial emergence in late December 2019. Published studies highlight the inequity and disparity in COVID-19 infections and deaths. Data collected globally and research data indicate that African and Black populations are more likely to contract and die from COVID-19 [5,6,9,31].

Anti-Black racism and colonial institutions contribute to disparities in access to health care, employment, education, housing, and physical environments, and food insecurity [32,33]. These determinants have been shown to have an impact on COVID-19 infection [14,16,34]. Through this scoping review, we will employ a decolonizing, African feminist, Black resistance lens to investigate the impact of COVID-19 on heterogeneous and intersectional Black communities in Canada and throughout the world. We will also explore the various forms of resistance that Black communities have established and employed during the global COVID-19 pandemic.

Our findings will be disseminated as peer-reviewed manuscripts and at conferences and student rounds, and could be of interest to government health agencies and organizations serving African/Black communities.

### Strengths

The exhaustive search strategy across numerous global databases is one of the strengths of this review. Another strength is the innovative research topic that examines the global impact of COVID-19 on African/Black communities.

### Limitations

One limitation of this scoping review is the wide range of inclusion criteria. Thus, the hand-searching of the literature by the research team could lead to personal interpretation of the criteria. Furthermore, the search was not translated into the 4 inclusion languages, and studies could be missed because of this. Moreover, it is generally understood that scoping reviews are not intended to be exhaustive, especially compared to systematic reviews and meta-analyses [35]. Still, the lack of critical appraisal and possibility of missing relevant studies have been reported as key challenges in conducting scoping reviews [36,37]. However, a narrative synthesis was found to be appropriate, which is both widely used and recommended in guidelines for scoping reviews. Another limitation to consider is associated with the differences and nuances in terminology across languages and regions. For example, systemic and structural racism may be described differently in different native languages using different terms. This may lead to a bias due to the lack of sufficient locally published articles in non-English journals in this review.

### Implications

This review will collect important data and evidence on African/Black communities related to COVID-19. Most importantly, the findings of this review could be used in decision-making for health policy and promotion, and could influence the services provided by health care facilities and community organizations around the globe that serve individuals from African and Black communities and help mitigate COVID-19 risk and ameliorate health outcomes and trajectories. In addition, the findings could offer guidance for future initiatives and emerging needs.

## Appendix

Multimedia Appendix 1 Preferred Reporting Items for Systematic Reviews and Meta-Analyses extension for Scoping Reviews (PRISMA-ScR) checklist.

Multimedia Appendix 2 Detailed search strategies for all databases.

## Abbreviations

PRISMA-ScR: Preferred Reporting Items for Systematic Reviews and Meta-Analyses extension for Scoping Reviews
