# Supplementary material for: The National and Global Impact of Systemic and Structural Violence on the Effective Prevention, Treatment, and Management of COVID-19 in African or Black Communities: Protocol for a Scoping Review
Source: JMIR Res Protoc. 2022 Oct 17;11(10):e40381. doi: 10.2196/40381 (PMC9578518; doi:10.2196/40381)
Supplement: Multimedia Appendix 2 [file resprot_v11i10e40381_app2.pdf]

Detailed search strategies for all databases

Search Strategies Designed By: Julia Martyniuk

Searches were run in August 2021. The strategies below correspond with the searches conducted in August 2021.

Database(s): **Ovid MEDLINE: Epub Ahead of Print, In-Process & Other Non-Indexed Citations, Ovid MEDLINE® Daily and Ovid MEDLINE®** 1946-Present
Search Strategy:

#	Searches	Results
1	exp Coronavirus/	87181
2	exp Coronavirus Infections/	106533
3	(coronavirus* or corona virus* or OC43 or NL63 or 229E or HKU1 or HCoV* or ncov* or covid* or sars-cov* or sarscov* or Sars$coronavirus* or Severe Acute Respiratory Syndrome Coronavirus* or 2019$nCov or Severe Acute Respiratory Syndrome Corona Virus).tw,kf,ot.	173610
4	((novel or new or nouveau) adj2 (CoV or nCoV or covid* or coronavirus* or corona virus or Pandemi*)).tw,kf,ot.	14611
5	((Wuhan or Hubei) adj5 pneumonia).tw,kf,ot.	355
6	((new or novel or "19" or "2019" or Wuhan or Hubei or China or Chinese) adj3 (coronavirus* or corona virus* or betacoronavirus* or CoV or HCoV)).tw,kf,ot.	48596
7	((coronavirus* or corona virus* or betacoronavirus*) adj3 (pandemic* or epidemic* or outbreak* or crisis)).tw,kf,ot.	8997
8	1 or 2 or 3 or 4 or 5 or 6 or 7	185346
9	limit 8 to yr="2019 -Current"	166753
10	exp african continental ancestry group/ or ethnic groups/	150584
11	Minority Groups/	15130
12	Minority Health/	839
13	(people of colo$r or person* of colo$r or POC or BAME or BIPOC or ((african* or afro*) adj5 (americ* or canad* or asia* or caribbean* or australi* or european* or brazil* or minorit* or refugee or migrant* or immigrant* or ancest* or native* or hispanic* or latin* or indigenous* or diaspora* or communit* or descen* or provider* or nurse* or doctor* or worker* or service user* or patient* or front line* or frontline* or people* or man or men or wom$n or race or population* or person* or individual* or group* or female* or male*))).tw,kf.	97176
14	((black or blacks) adj5 (americ* or canad* or asia* or caribbean* or australi* or european* or brazil* or minorit* or refugee or migrant* or immigrant* or ancest* or native* or hispanic* or latin* or indigenous* or diaspora* or communit* or descen* or provider* or nurse* or doctor* or	57326

	worker* or service user* or patient* or front line* or frontline* or people* or man or men or wom$n or race or population* or person* or individual* or group* or female* or male*)).tw,kf.	
15	(((ethnic* or racial* or race) adj5 (group* or minorit* or disparit* or divers* or equal* or inequal* or discriminat*)) or mixed race or mixed racial* or multi racial* or mutli race or multiracial* or multirace).tw,kf.	87613
16	10 or 11 or 12 or 13 or 14 or 15	286281
17	prejudice/ or racism/	28679
18	(racism or racist* or racial* or anti-black* or antiblack* or structural violence* or systemic violence*).tw,kf.	54130
19	(white supremac* or white hegemon*).tw,kf.	95
20	(prejudice* or discriminat* or intolerance* or oppress* or bias* or hostil*).tw,kf.	550272
21	((structur* or institution* or systemic* or systematic* or generational* or intersect* or health*) adj5 (violence* or polic* or barrier* or disparit* or inequalit* or trauma*)).tw,kf.	148756
22	(decoloni* or de coloni* or anti oppress* or antioppress*).tw,kf.	1798
23	17 or 18 or 19 or 20 or 21 or 22	748632
24	9 and 16 and 23	1364

Database(s): **Embase Classic+Embase** 1947 to 2021 August 03
Search Strategy:

#	Searches	Results
1	exp coronavirinae/	59331
2	exp Coronavirus infection/	154180
3	(coronavirus* or corona virus* or OC43 or NL63 or 229E or HKU1 or HCoV* or ncov* or covid* or sars-cov* or sarscov* or Sars$coronavirus* or Severe Acute Respiratory Syndrome Coronavirus* or 2019$nCov or Severe Acute Respiratory Syndrome Corona Virus).tw,kw,ot.	174597
4	((novel or new or nouveau) adj2 (CoV or nCoV or covid* or coronavirus* or corona virus or Pandemi*)).tw,kw,ot.	14799
5	((Wuhan or Hubei) adj5 pneumonia).tw,kw,ot.	393
6	((new or novel or "19" or "2019" or Wuhan or Hubei or China or Chinese) adj3 (coronavirus* or corona virus* or betacoronavirus* or CoV or HCoV)).tw,kw,ot.	46678
7	((coronavirus* or corona virus* or betacoronavirus*) adj3 (pandemic* or epidemic* or outbreak* or crisis)).tw,kw,ot.	8618
8	1 or 2 or 3 or 4 or 5 or 6 or 7	197115

9	limit 8 to yr="2019 -Current"	173394
10	exp black person/	122946
11	ethnic group/ or exp afro-asiatic people/ or exp negrito/ or exp niger-congo people/ or exp nilo-saharan people/ or exp pygmy/	92241
12	minority group/	16239
13	minority health/	896
14	(people of colo$r or person* of colo$r or POC or BAME or BIPOC or ((african* or afro*) adj5 (americ* or canad* or asia* or caribbean* or australi* or european* or brazil* or minorit* or refugee or migrant* or immigrant* or ancest* or native* or hispanic* or latin* or indigenous* or diaspora* or communit* or descen* or provider* or nurse* or doctor* or worker* or service user* or patient* or front line* or frontline* or people* or man or men or wom$n or race or population* or person* or individual* or group* or female* or male*))).tw,kw.	145807
15	((black or blacks) adj5 (americ* or canad* or asia* or caribbean* or australi* or european* or brazil* or minorit* or refugee or migrant* or immigrant* or ancest* or native* or hispanic* or latin* or indigenous* or diaspora* or communit* or descen* or provider* or nurse* or doctor* or worker* or service user* or patient* or front line* or frontline* or people* or man or men or wom$n or race or population* or person* or individual* or group* or female* or male*)).tw,kw.	82471
16	(((ethnic* or racial* or race) adj5 (group* or minorit* or disparit* or divers* or equal* or inequal* or discriminat*)) or mixed race or mixed racial* or multi racial* or mutli race or multiracial* or multirace).tw,kw.	116155
17	10 or 11 or 12 or 13 or 14 or 15 or 16	379519
18	prejudice/	3433
19	exp racism/	8799
20	exp race relation/	9629
21	(racism or racist* or racial* or anti-black* or antiblack* or structural violence* or systemic violence*).tw,kw.	72256
22	(white supremac* or white hegemon*).tw,kw.	92
23	(prejudice* or discriminat* or intolerance* or oppress* or bias* or hostil*).tw,kw.	701354
24	((structur* or institution* or systemic or systematic* or generational* or intersect* or health*) adj5 (violence* or polic* or barrier* or disparit* or inequalit* or trauma)).tw,kw.	175014
25	(decoloni* or de coloni* or anti oppress* or antioppress*).tw,kw.	2291
26	18 or 19 or 20 or 21 or 22 or 23 or 24 or 25	929612
27	9 and 17 and 26	1569

Database(s): **APA PsycInfo** 1806 to July Week 4 2021
Search Strategy:

#	Searches	Results
1	exp coronavirus/	3019
2	(coronavirus* or corona virus* or OC43 or NL63 or 229E or HKU1 or HCoV* or ncov* or covid* or sars-cov* or sarscov* or Sars$coronavirus* or Severe Acute Respiratory Syndrome Coronavirus* or 2019$nCov or Severe Acute Respiratory Syndrome Corona Virus).tw,ot.	7759
3	((novel or new or nouveau) adj2 (CoV or nCoV or covid* or coronavirus* or corona virus or Pandemi*)).tw,ot.	536
4	((Wuhan or Hubei) adj5 pneumonia).tw,ot.	7
5	((new or novel or "19" or "2019" or Wuhan or Hubei or China or Chinese) adj3 (coronavirus* or corona virus* or betacoronavirus* or CoV or HCoV)).tw,ot.	1564
6	((coronavirus* or corona virus* or betacoronavirus*) adj3 (pandemic* or epidemic* or outbreak* or crisis)).tw,ot.	688
7	1 or 2 or 3 or 4 or 5 or 6	8018
8	limit 7 to yr="2019 -Current"	7721
9	"racial and ethnic groups"/ or exp african cultural groups/ or exp blacks/ or exp multiracial/	69524
10	minority groups/	16220
11	(people of colo$r or person* of colo$r or POC or BAME or BIPOC or ((african* or afro*) adj5 (americ* or canad* or asia* or caribbean* or australi* or european* or brazil* or minorit* or refugee or migrant* or immigrant* or ancest* or native* or hispanic* or latin* or indigenous* or diaspora* or communit* or descen* or provider* or nurse* or doctor* or worker* or service user* or patient* or front line* or frontline* or people* or man or men or wom$n or race or population* or person* or individual* or group* or female* or male*))).tw.	61115
12	((black or blacks) adj5 (americ* or canad* or asia* or caribbean* or australi* or european* or brazil* or minorit* or refugee or migrant* or immigrant* or ancest* or native* or hispanic* or latin* or indigenous* or diaspora* or communit* or descen* or provider* or nurse* or doctor* or worker* or service user* or patient* or front line* or frontline* or people* or man or men or wom$n or race or population* or person* or individual* or group* or female* or male*)).tw.	35222
13	(((ethnic* or racial* or race) adj5 (group* or minorit* or disparit* or divers* or equal* or inequal* or discriminat*)) or mixed race or mixed racial* or multi racial* or mutli race or multiracial* or multirace).tw.	63285
14	9 or 10 or 11 or 12 or 13	156183
15	prejudice/ or exp hate crimes/ or exp implicit bias/ or exp "race and ethnic discrimination"/ or exp racial bias/ or exp racism/ or exp stereotyped attitudes/	34201
16	racism/ or exp racial disparities/ or exp social discrimination/	21284

17	(racism or racist* or racial* or anti-black* or antiblack* or structural violence* or systemic violence*).tw.	64465
18	(white supremac* or white hegemon*).tw.	686
19	(prejudice* or discriminat* or intolerance* or oppress* or bias* or hostil*).tw.	262994
20	((structur* or institution* or systemic or systematic* or generational* or intersect* or health*) adj5 (violence* or polic* or barrier* or disparit* or inequalit* or trauma)).tw.	66557
21	(decoloni* or de coloni* or anti oppress* or antioppress*).tw.	1808
22	15 or 16 or 17 or 18 or 19 or 20 or 21	381374
23	8 and 14 and 22	201

Database(s): **CAB Abstracts** 1973 to 2021 Week 30
Search Strategy:

#	Searches	Results
1	(coronavirus* or corona virus* or OC43 or NL63 or 229E or HKU1 or HCoV* or ncov* or covid* or sars-cov* or sarscov* or Sars$coronavirus* or Severe Acute Respiratory Syndrome Coronavirus* or 2019$nCov or Severe Acute Respiratory Syndrome Corona Virus).mp.	26700
2	((novel or new or nouveau) adj2 (CoV or nCoV or covid* or coronavirus* or corona virus or Pandemi*)).mp.	2134
3	((Wuhan or Hubei) adj5 pneumonia).mp.	58
4	((new or novel or "19" or "2019" or Wuhan or Hubei or China or Chinese) adj3 (coronavirus* or corona virus* or betacoronavirus* or CoV or HCoV)).mp.	11764
5	((coronavirus* or corona virus* or betacoronavirus*) adj3 (pandemic* or epidemic* or outbreak* or crisis)).mp.	1232
6	1 or 2 or 3 or 4 or 5	26992
7	limit 6 to yr="2019 -Current"	16169
8	exp african americans/ or exp ethnic groups/	32603
9	minorities/	2543
10	(people of colo$r or person* of colo$r or POC or BAME or BIPOC or ((african* or afro*) adj5 (americ* or canad* or asia* or caribbean* or australi* or european* or brazil* or minorit* or refugee or migrant* or immigrant* or ancest* or native* or hispanic* or latin* or indigenous* or diaspora* or communit* or descen* or provider* or nurse* or doctor* or worker* or service user* or patient* or front line* or frontline* or people* or man or men or wom$n or race or population* or person* or individual* or group* or female* or male*))).mp.	26826

11	((black or blacks) adj5 (americ* or canad* or asia* or caribbean* or australi* or european* or brazil* or minorit* or refugee or migrant* or immigrant* or ancest* or native* or hispanic* or latin* or indigenous* or diaspora* or communit* or descen* or provider* or nurse* or doctor* or worker* or service user* or patient* or front line* or frontline* or people* or man or men or wom$n or race or population* or person* or individual* or group* or female* or male*)).mp.	21867
12	(((ethnic* or racial* or race) adj5 (group* or minorit* or disparit* or divers* or equal* or inequal* or discriminat*)) or mixed race or mixed racial* or multi racial* or mutli race or multiracial* or multirace).mp.	32095
13	8 or 9 or 10 or 11 or 12	77007
14	discrimination/ or exp racial discrimination/ or exp health inequalities/	8679
15	race relations/	1286
16	(racism or racist* or racial* or anti-black* or antiblack* or structural violence* or systemic violence*).mp.	7342
17	(white supremac* or white hegemon*).mp.	45
18	(prejudice* or discriminat* or intolerance* or oppress* or bias* or hostil*).mp.	133991
19	((structur* or institution* or systemic or systematic* or generational* or intersect* or health*) adj5 (violence* or polic* or barrier* or disparit* or inequalit* or trauma)).mp.	57876
20	(decoloni* or de coloni* or anti oppress* or antioppress*).mp.	710
21	14 or 15 or 16 or 17 or 18 or 19 or 20	196543
22	7 and 13 and 21	131

Database: **Cochrane Library**

ID Search Hits
#1 MeSH descriptor: [Coronavirus] explode all trees 397
#2 MeSH descriptor: [Coronavirus Infections] explode all trees 1053
#3 (coronavirus* or "corona virus*" or OC43 or NL63 or 229E or HKU1 or HCoV* or ncov* or covid* or sars-cov* or sarscov* or "Sars$coronavirus*" or "Severe Acute Respiratory Syndrome Coronavirus*" or 2019$nCov or "Severe Acute Respiratory Syndrome Corona Virus"):ti,ab,kw 7304
#4 ((novel or new or nouveau) NEAR/2 (CoV or nCoV or covid* or coronavirus* or "corona virus" or Pandemi*)):ti,ab,kw 639
#5 ((Wuhan or Hubei) NEAR/5 pneumonia):ti,ab,kw 23
#6 ((new or novel or "19" or "2019" or Wuhan or Hubei or China or Chinese) NEAR/3 (coronavirus* or "corona virus*" or betacoronavirus* or CoV or HCoV)):ti,ab,kw 2784
#7 ((coronavirus* or "corona virus*" or betacoronavirus*) NEAR/3 (pandemic* or epidemic* or outbreak* or crisis)):ti,ab,kw 182
#8 #1 OR #2 OR #3 OR #4 OR #5 OR #6 OR #7 7345
#9 #8 with Cochrane Library publication date Between Nov 2019 and Dec 2021 7042
#10 MeSH descriptor: [African Continental Ancestry Group] explode all trees 3028

#11 MeSH descriptor: [Ethnic Groups] this term only 879
#12 MeSH descriptor: [Minority Groups] this term only 366
#13 MeSH descriptor: [Minority Health] this term only 25
#14 ("people of colo?r" or "person* of colo?r" or POC or BAME or BIPOC or ((african* or afro*) NEAR/5 (americ* or canad* or asia* or caribbean* or australi* or european* or brazil* or minorit* or refugee or migrant* or immigrant* or ancest* or native* or hispanic* or latin* or indigenous* or diaspora* or communit* or descen* or provider* or nurse* or doctor* or worker* or "service user*" or patient* or "front line*" or frontline* or people* or man or men or wom?n or race or population* or person* or individual* or group* or female* or male*))):ti,ab,kw 12175
#15 ((black or blacks) NEAR/5 (americ* or canad* or asia* or caribbean* or australi* or european* or brazil* or minorit* or refugee or migrant* or immigrant* or ancest* or native* or hispanic* or latin* or indigenous* or diaspora* or communit* or descen* or provider* or nurse* or doctor* or worker* or "service user*" or patient* or "front line*" or frontline* or people* or man or men or wom?n or race or population* or person* or individual* or group* or female* or male*)):ti,ab,kw 4779
#16 (((ethnic* or racial* or race) NEAR/5 (group* or minorit* or disparit* or divers* or equal* or inequal* or discriminat*)) or "mixed race" or "mixed racial*" or "multi racial*" or "mutli race" or multiracial* or multirace):ti,ab,kw 6414
#17 #10 OR #11 OR #12 OR #13 OR #14 OR #15 OR #16 20123
#18 MeSH descriptor: [Prejudice] this term only 288
#19 MeSH descriptor: [Racism] this term only 37
#20 (racism or racist* or racial* or "anti-black*" or antiblack* or "structural violence*" or "systemic violence*"):ti,ab,kw 3044
#21 ("white supremac*" or "white hegemon*"):ti,ab,kw 0
#22 (prejudice* or discriminat* or intolerance* or oppress* or bias* or hostil*):ti,ab,kw 38924
#23 ((structur* or institution* or systemic or systematic* or generational* or intersect* or health*) NEAR/5 (violence* or polic* or barrier* or disparit* or inequalit* or trauma)):ti,ab,kw 7054
#24 (decoloni* or "de coloni*" or "anti oppress*" or antioppress*):ti,ab,kw 303
#25 #18 OR #19 OR #20 OR #21 OR #22 OR #23 OR #24 48166
#26 #9 AND #17 AND #25 47

Database: **EBSCO CINAHL**

#	Query	Limiters/Expanders	Last Run Via	Results
S22	S8 AND S14 AND S21	Search modes - Boolean/Phrase	Interface - EBSCOhost Research Databases<br>Search Screen - Advanced Search<br>Database - CINAHL Plus with Full Text	761
S21	S15 OR S16 OR S17 OR S18 OR S19 OR S20	Search modes - Boolean/Phrase	Interface - EBSCOhost Research Databases	231,628

			Search Screen - Advanced Search<br>Database - CINAHL Plus with Full Text	
S20	TI ((decoloni* or "de coloni*" or "anti oppress*" or antioppress*)) OR AB ((decoloni* or "de coloni*" or "anti oppress*" or antioppress*))	Search modes - Boolean/Phrase	Interface - EBSCOhost Research Databases<br>Search Screen - Advanced Search<br>Database - CINAHL Plus with Full Text	1,126
S19	TI (((structur* or institution* or systemic* or systematic* or generational* or intersect* or health*) N5 (violence* or polic* or barrier* or disparit* or inequalit* or trauma*))) OR AB (((structur* or institution* or systemic* or systematic* or generational* or intersect* or health*) N5 (violence* or polic* or barrier* or disparit* or inequalit* or trauma*)))	Search modes - Boolean/Phrase	Interface - EBSCOhost Research Databases<br>Search Screen - Advanced Search<br>Database - CINAHL Plus with Full Text	86,531
S18	TI ((prejudice* or discriminat* or intolerance* or oppress* or bias* or hostil*)) OR AB ((prejudice* or discriminat* or intolerance* or oppress* or bias* or hostil*))	Search modes - Boolean/Phrase	Interface - EBSCOhost Research Databases<br>Search Screen - Advanced Search<br>Database - CINAHL Plus with Full Text	120,458
S17	TI (("white supremac*" or "white hegemon*")) OR AB (("white supremac*" or "white hegemon*"))	Search modes - Boolean/Phrase	Interface - EBSCOhost Research Databases<br>Search Screen - Advanced Search<br>Database - CINAHL Plus with Full Text	84

S16	TI ((racism or racist* or racial* or "anti-black*" or antiblack* or "structural violence*" or "systemic violence*")) OR AB ((racism or racist* or racial* or "anti-black*" or antiblack* or "structural violence*" or "systemic violence*"))	Search modes - Boolean/Phrase	Interface - EBSCOhost Research Databases<br>Search Screen - Advanced Search<br>Database - CINAHL Plus with Full Text	29,620
S15	(MH "Prejudice") OR (MH "Racial Equality") OR (MH "Racism")	Search modes - Boolean/Phrase	Interface - EBSCOhost Research Databases<br>Search Screen - Advanced Search<br>Database - CINAHL Plus with Full Text	13,889
S14	S9 OR S10 OR S11 OR S12 OR S13	Search modes - Boolean/Phrase	Interface - EBSCOhost Research Databases<br>Search Screen - Advanced Search<br>Database - CINAHL Plus with Full Text	135,738
S13	TI ((((ethnic* or racial* or race) N5 (group* or minorit* or disparit* or divers* or equal* or inequal* or discriminat*)) or "mixed race" or "mixed racial*" or "multi racial*" or "mutli race" or multiracial* or multirace)) OR AB ((((ethnic* or racial* or race) N5 (group* or minorit* or disparit* or divers* or equal* or inequal* or discriminat*)) or "mixed race" or "mixed racial*" or "multi racial*" or "mutli race" or multiracial* or multirace))	Search modes - Boolean/Phrase	Interface - EBSCOhost Research Databases<br>Search Screen - Advanced Search<br>Database - CINAHL Plus with Full Text	39,816

S12	TI (((black or blacks) N5 (americ* or canad* or asia* or caribbean* or australi* or european* or brazil* or minorit* or refugee or migrant* or immigrant* or ancest* or native* or hispanic* or latin* or indigenous* or diaspora* or communit* or descen* or provider* or nurse* or doctor* or worker* or "service user*" or patient* or "front line*" or frontline* or people* or man or men or wom#n or race or population* or person* or individual* or group* or female* or male*))) OR AB (((black or blacks) N5 (americ* or canad* or asia* or caribbean* or australi* or european* or brazil* or minorit* or refugee or migrant* or immigrant* or ancest* or native* or hispanic* or latin* or indigenous* or diaspora* or communit* or descen* or provider* or nurse* or doctor* or worker* or "service user*" or patient* or "front line*" or frontline* or people* or man or men or wom#n or race or population* or person* or individual* or group* or female* or male*)))	Search modes - Boolean/Phrase	Interface - EBSCOhost Research Databases<br>Search Screen - Advanced Search<br>Database - CINAHL Plus with Full Text	26,501

S11	TI (("people of col#r" or "person* of col#r" or POC or BAME or BIPOC or ((african* or afro*) N5 (americ* or canad* or asia* or caribbean* or australi* or european* or brazil* or minorit* or refugee or migrant* or immigrant* or ancest* or native* or hispanic* or latin* or indigenous* or diaspora* or communit* or descen* or provider* or nurse* or doctor* or worker* or "service user*" or patient* or "front line*" or frontline* or people* or man or men or wom$n or race or population* or person* or individual* or group* or female* or male*)))) OR AB (("people of col#r" or "person* of col#r" or POC or BAME or BIPOC or ((african* or afro*) N5 (americ* or canad* or asia* or caribbean* or australi* or european* or brazil* or minorit* or refugee or migrant* or immigrant* or ancest* or native* or hispanic* or latin* or indigenous* or diaspora* or communit* or descen* or provider* or nurse* or doctor* or worker* or "service user*" or patient* or "front line*" or frontline* or people* or man or men or wom#n or race or population* or person* or individual* or	Search modes - Boolean/Phrase	Interface - EBSCOhost Research Databases<br>Search Screen - Advanced Search<br>Database - CINAHL Plus with Full Text	42,120

	group* or female* or male*))))			
S10	(MH "Health Personnel, Minority+") OR (MH "Nurses, Minority")	Search modes - Boolean/Phrase	Interface - EBSCOhost Research Databases<br>Search Screen - Advanced Search<br>Database - CINAHL Plus with Full Text	1,452
S9	(MH "Minority Groups") OR (MH "Ethnic Groups") OR (MH "Black Persons")	Search modes - Boolean/Phrase	Interface - EBSCOhost Research Databases<br>Search Screen - Advanced Search<br>Database - CINAHL Plus with Full Text	90,064
S8	S1 OR S2 OR S3 OR S4 OR S5 OR S6	Limiters - Published Date: 20191101-20211231<br>Search modes - Boolean/Phrase	Interface - EBSCOhost Research Databases<br>Search Screen - Advanced Search<br>Database - CINAHL Plus with Full Text	57,694
S7	S1 OR S2 OR S3 OR S4 OR S5 OR S6	Search modes - Boolean/Phrase	Interface - EBSCOhost Research Databases<br>Search Screen - Advanced Search<br>Database - CINAHL Plus with Full Text	61,251
S6	TI (((coronavirus* or "corona virus*" or betacoronavirus*) N3 (pandemic* or epidemic* or outbreak* or crisis))) OR AB (((coronavirus* or	Search modes - Boolean/Phrase	Interface - EBSCOhost Research Databases<br>Search Screen - Advanced Search<br>Database - CINAHL Plus with Full Text	5,832

	"corona virus*" or betacoronavirus*) N3 (pandemic* or epidemic* or outbreak* or crisis)))			
S5	TI (((new or novel or "19" or "2019" or Wuhan or Hubei or China or Chinese) N3 (coronavirus* or "corona virus*" or betacoronavirus* or CoV or HCoV))) OR AB (((new or novel or "19" or "2019" or Wuhan or Hubei or China or Chinese) N3 (coronavirus* or "corona virus*" or betacoronavirus* or CoV or HCoV)))	Search modes - Boolean/Phrase	Interface - EBSCOhost Research Databases<br>Search Screen - Advanced Search<br>Database - CINAHL Plus with Full Text	10,859
S4	TI (((Wuhan or Hubei) N5 pneumonia)) OR AB (((Wuhan or Hubei) N5 pneumonia))	Search modes - Boolean/Phrase	Interface - EBSCOhost Research Databases<br>Search Screen - Advanced Search<br>Database - CINAHL Plus with Full Text	126
S3	TI (((novel or new or nouveau) N2 (CoV or nCoV or covid* or coronavirus* or "corona virus" or Pandemi*))) OR AB (((novel or new or nouveau) N2 (CoV or nCoV or covid* or coronavirus* or "corona virus" or Pandemi*)))	Search modes - Boolean/Phrase	Interface - EBSCOhost Research Databases<br>Search Screen - Advanced Search<br>Database - CINAHL Plus with Full Text	3,911
S2	TI ((coronavirus* or "corona virus*" or OC43 or NL63 or 229E or HKU1 or HCoV* or ncov* or covid* or "sars-cov*" or sarscov* or “Sars?coronavirus*” or "Severe Acute	Search modes - Boolean/Phrase	Interface - EBSCOhost Research Databases<br>Search Screen - Advanced Search<br>Database - CINAHL Plus with Full Text	54,907

	Respiratory Syndrome Coronavirus*" or “2019?nCov” or "Severe Acute Respiratory Syndrome Corona Virus")) OR AB ((coronavirus* or "corona virus*" or OC43 or NL63 or 229E or HKU1 or HCoV* or ncov* or covid* or "sars-cov*" or sarscov* or “Sars?coronavirus*” or "Severe Acute Respiratory Syndrome Coronavirus*" or “2019?nCov” or "Severe Acute Respiratory Syndrome Corona Virus"))			
S1	(MH "Coronavirus+") OR (MH "Coronavirus Infections+")	Search modes - Boolean/Phrase	Interface - EBSCOhost Research Databases<br>Search Screen - Advanced Search<br>Database - CINAHL Plus with Full Text	29,890

Database: **Scopus**

((TITLE-ABS-KEY ((coronavirus* OR "corona virus*" OR oc43 OR nl63 OR 229e OR hku1 OR hcov* OR ncov* OR covid* OR "sars-cov*" OR sarscov* OR "Sars?coronavirus*" OR "Severe Acute Respiratory Syndrome Coronavirus*" OR "2019?nCov" OR "Severe Acute Respiratory Syndrome Corona Virus")) OR TITLE-ABS-KEY (((novel OR new OR nouveau) W/2 (cov OR ncov OR covid* OR coronavirus* OR "corona virus" OR pandemi*))) OR TITLE-ABS-KEY (((wuhan OR hubei) W/5 pneumonia)) OR TITLE-ABS-KEY (((new OR novel OR "19" OR "2019" OR wuhan OR hubei OR china OR chinese) W/3 (coronavirus* OR "corona virus*" OR betacoronavirus* OR cov OR hcov))) OR TITLE-ABS-KEY (((coronavirus* OR "corona virus*" OR betacoronavirus*) W/3 (pandemic* OR epidemic* OR outbreak* OR crisis))))) AND ((TITLE-ABS-KEY (("people of colo?r" OR "person* of colo?r" OR poc OR bame OR bipoc OR ((african* OR afro*) W/5 (americ* OR canad* OR asia* OR caribbean* OR australi* OR european* OR brazil* OR minorit* OR refugee OR migrant* OR immigrant* OR ancest* OR native* OR hispanic* OR latin* OR indigenous* OR diaspora* OR communit* OR descen* OR provider* OR nurse* OR doctor* OR worker* OR "service user*" OR patient* OR "front line*" OR frontline* OR people* OR man OR men OR wom?n OR race OR population* OR person* OR individual* OR group* OR female* OR male*)))) OR TITLE-ABS-KEY (((black OR blacks) W/5 (americ* OR canad* OR asia* OR caribbean* OR australi* OR european* OR brazil* OR minorit* OR refugee OR migrant* OR immigrant* OR ancest* OR native* OR hispanic* OR latin* OR indigenous* OR diaspora* OR communit* OR descen* OR provider* OR nurse* OR doctor* OR worker* OR "service user*" OR patient* OR "front line*" OR frontline* OR people* OR man OR men OR wom?n OR race OR population* OR person* OR individual* OR group* OR female* OR male*))) OR TITLE-ABS-KEY ((((ethnic* OR racial* OR race) W/5 (group* OR minorit* OR disparit* OR divers* OR equal* OR inequal* OR discriminat*)) OR "mixed race" OR "mixed racial*" OR "multi racial*" OR "mutli race" OR multiracial* OR multirace)))) AND ((TITLE-ABS-KEY ((racism OR racist* OR racial* OR "anti-black*" OR antiblack* OR "structural violence*" OR "systemic violence*")) OR TITLE-ABS-KEY (("white supremac*" OR "white hegemon*")) OR TITLE-ABS-KEY ((prejudice* OR discriminat* OR intolerance* OR oppress* OR bias* OR hostil*)) OR TITLE-ABS-KEY (((structur* OR institution* OR systemic* OR systematic* OR generational* OR intersect* OR health*) W/5 (violence* OR polic* OR barrier* OR disparit* OR inequalit* OR trauma*))) OR TITLE-ABS-KEY ((decoloni* OR "de coloni*" OR "anti oppress*" OR antioppress*)))) AND (LIMIT-TO (PUBYEAR , 2022) OR LIMIT-TO (PUBYEAR , 2021) OR LIMIT-TO (PUBYEAR , 2020) OR LIMIT-TO (PUBYEAR , 2019))

Database: **Web of Science**

(coronavirus* or "corona virus*" or OC43 or NL63 or 229E or HKU1 or HCoV* or ncov* or covid* or "sars-cov*" or sarscov* or "Sars$coronavirus*" or "Severe Acute Respiratory Syndrome Coronavirus*" or "2019$nCov" or "Severe Acute Respiratory Syndrome Corona Virus") (Topic) or ((novel or new or nouveau) NEAR/2 (CoV or nCoV or covid* or coronavirus* or "corona virus" or Pandemi*)) (Topic) or ((Wuhan or Hubei) NEAR/5 pneumonia) (Topic) or ((new or novel or "19" or "2019" or Wuhan or Hubei or China or Chinese) NEAR/3 (coronavirus* or "corona virus*" or betacoronavirus* or CoV or HCoV)) (Topic) or ((coronavirus* or "corona virus*" or betacoronavirus*) NEAR/3 (pandemic* or epidemic* or outbreak* or crisis)) (Topic)

AND

("people of colo$r" or "person* of colo$r" or POC or BAME or BIPOC or ((african* or afro*) NEAR/5 (americ* or canad* or asia* or caribbean* or australi* or european* or brazil* or minorit* or refugee or migrant* or immigrant* or ancest* or native* or hispanic* or latin* or indigenous* or diaspora* or communit* or descen* or provider* or nurse* or doctor* or worker* or "service user*" or patient* or "front line*" or frontline* or people* or man or men or wom$n or race or population* or person* or individual* or group* or female* or male*))) (Topic) or ((black or blacks) NEAR/5 (americ* or canad* or asia* or caribbean* or australi* or european* or brazil* or minorit* or refugee or migrant* or immigrant* or ancest* or native* or hispanic* or latin* or indigenous* or diaspora* or communit* or descen* or provider* or nurse*

or doctor* or worker* or "service user*" or patient* or "front line*" or frontline* or people* or man or men or wom$n or race or population* or person* or individual* or group* or female* or male*)) (Topic) or (((ethnic* or racial* or race) NEAR/5 (group* or minorit* or disparit* or divers* or equal* or inequal* or discriminat*)) or "mixed race" or "mixed racial*" or "multi racial*" or "mutli race" or multiracial* or multirace) (Topic)
AND
(racism or racist* or racial* or "anti-black*" or antiblack* or "structural violence*" or "systemic violence*") (Topic) or ("white supremac*" or "white hegemon*") (Topic) or (prejudice* or discriminat* or intolerance* or oppress* or bias* or hostil*) (Topic) or ((structur* or institution* or systemic* or systematic* or generational* or intersect* or health*) NEAR/5 (violence* or polic* or barrier* or disparit* or inequalit* or trauma*)) (Topic) or (decoloni* or "de coloni*" or "anti oppress*" or antioppress*) (Topic)
AND
2021 or 2020 or 2019 (Publication Years)

Database: **Global Index Medicus**

tw:((af:(tw:(tw:((af:(mh:"Coronavirus" OR mh:"Coronavirus Infections")) OR (tw:((coronavirus* OR "corona virus" OR oc43 OR nl63 OR 229e OR hku1 OR hcov* OR ncov* OR covid* OR sars-cov* OR sarscov* OR sars*coronavirus* OR "Severe Acute Respiratory Syndrome Coronavirus" OR 2019*ncov OR "Severe Acute Respiratory Syndrome Corona Virus"))) OR (tw:(((novel OR new OR nouveau) AND (cov OR ncov OR covid* OR coronavirus* OR "corona virus" OR pandemi*)))) OR (tw:(((wuhan OR hubei) AND pneumonia))) OR (tw:(((new OR novel OR "19" OR "2019" OR wuhan OR hubei OR china OR chinese) AND (coronavirus* OR "corona virus" OR betacoronavirus* OR cov OR hcov)))) OR (tw:(((coronavirus* OR "corona virus" OR betacoronavirus*) AND (pandemic* OR epidemic* OR outbreak* OR crisis)))))))) AND (af:(tw:((af:(mh:m01.686.508.100* OR mh:"ethnic groups" OR mh:"Minority Groups" OR mh:minority health)) OR (tw:(("people of color" OR "people of colour" OR "person of color" OR "persons of color" OR "person of colour" OR "persons of colour" OR poc OR bame OR bipoc OR ((african* OR afro*) AND (americ* OR canad* OR asia* OR caribbean* OR australi* OR european* OR brazil* OR minorit* OR refugee OR migrant* OR immigrant* OR ancest* OR native* OR hispanic* OR latin* OR indigenous* OR diaspora* OR communit* OR descen* OR provider* OR nurse* OR doctor* OR worker* OR "service user" OR "service users" OR patient* OR "front line" OR frontline* OR people* OR man OR men OR wom*n OR race OR population* OR person* OR individual* OR group* OR female* OR male*))))) OR (tw:(((black OR blacks) AND (americ* OR canad* OR asia* OR caribbean* OR australi* OR european* OR brazil* OR minorit* OR refugee OR migrant* OR immigrant* OR ancest* OR native* OR hispanic* OR latin* OR indigenous* OR diaspora* OR communit* OR descen* OR provider* OR nurse* OR doctor* OR worker* OR "service user" OR "service users" OR patient* OR "front line" OR frontline* OR people* OR man OR men OR wom*n OR race OR population* OR person* OR individual* OR group* OR female* OR male*)))) OR (tw:((((ethnic* OR racial* OR race) AND (group* OR minorit* OR disparit* OR divers* OR equal* OR inequal* OR discriminat*)) OR "mixed race" OR "mixed racial" OR "multi racial" OR "mutli race" OR multiracial* OR multirace)))))) AND (af:(tw:(tw:((af:(tw:((af:(mh:"prejudice" OR mh:"racism")) OR (tw:((racism OR racist* OR racial* OR anti-black* OR antiblack* OR "structural violence" OR "systemic violence"))) OR (tw:((prejudice* OR discriminat* OR intolerance* OR oppress* OR bias* OR hostil*))) OR (tw:((decoloni* OR de-coloni* OR anti-oppress* OR antioppress*))) OR (tw:(((structur* OR institution* OR systemic* OR systematic* OR generational* OR intersect* OR health*) AND (violence* OR polic* OR barrier* OR disparit* OR inequalit* OR trauma*))))))) OR (tw:(("white supremacy" OR "white supremist" OR "white hegemony" OR "white hegemonic"))))))))
